# Grass pollen nasal challenge is associated with increases in Th2 cytokines, Eotaxin, MDC and IL-6 in nasal fluid

**DOI:** 10.1186/2045-7022-3-S2-P29

**Published:** 2013-07-16

**Authors:** Guy Scadding, Aarif Eifan, Martin Penagos, Gitte Konsgaard Koed, Peter Adler Wurtzen, Mohamed Shamji, Stephen Durham

**Affiliations:** 1 Imperial College, London, UK; 2 Imperial College, London, Allergy and Clinical Immunology, London, UK; 3 ALK-Abello, Vaccine Research and Discovery, Hørsholm, Denmark

## Background

We previously validated a grass pollen nasal challenge model to record clinical outcomes and local biomarkers in nasal fluid [1]. Here we further validate our methods and compare the expression of Th2- and Th1-related cytokines, chemokines and IL-17.

## Methods

18 grass pollen allergics underwent nasal allergen challenges; 15 grass allergics had matched diluent challenges. Symptom scores and peak nasal inspiratory flow were recorded; nasal fluid was collected using polyurethane sponges, then extracted by centrifugation across microfilters. Fluid was then tested for Th2 cytokines by Mesoscale 7-plex multi-spot plate assay. A further 6 allergics underwent nasal allergen challenges with collection of fluid using both polyurethane sponges and Leukosorb filter strips; nasal fluid was analysed by Milliplex cytokine/chemokine magnetic bead multiplex assay.

## Results

Allergen vs diluent challenges; multi-spot plate assay: symptoms increased, and peak nasal flow decreased, following allergen but not diluent challenge (both p<0.001, between groups difference). Levels of IL-4 (p<0.01), IL-5 and IL-13 (both P<0.001) were maximally increased at 5 hours compared to pre-challenge; no significant increases were seen following diluent challenge. Between group differences (allergen vs diluent) for IL-4, -5 and -13 were seen at 4 and 6 hours (all p<0.01).Allergen challenge; magnetic bead assay: IL-5 was increased at 6 hours (p=0.03 vs pre-challenge), with IL-13 and IL-4 also showing a trend towards an increase (both p=0.06 vs pre-challenge). Eotaxin and MDC were increased at 6 hours (both p=0.03 vs pre-challenge); IL-6 was elevated at 2 hours (p=0.03 vs pre-challenge). Levels of IL-17A, IL-27, IL-23, IFN-gamma and IL-12p70 were low and did not change significantly after allergen challenge. High levels of IL-8 were detected, maximal at baseline, but did not change significantly after challenge. Polyurethane sponges proved superior to filters for all measurements.

## Conclusions

Grass pollen nasal challenge is associated with a strong local Th2, but not Th1, response detectable in nasal secretions. Despite elevation of IL-6, there is no significant local Th17 response, up to 6 hours. Responses are allergen-driven and independent of the intervention and any diurnal variation. Multiplex assays are capable of detecting cytokines and chemokines in low volumes (25mcl) of nasal fluid by both multi-spot and micro-bead techniques.
